# Finding ways to support radiographers as teachers

**DOI:** 10.1002/jmrs.399

**Published:** 2020-05-25

**Authors:** Andrea Thompson, Darci Taylor

**Affiliations:** ^1^ School of Medicine Deakin University Geelong Victoria Australia; ^2^ Centre for Medical and Health Sciences Education University of Auckland Auckland New Zealand; ^3^ CloudFirst Redesign Deakin University Geelong Victoria Australia

**Keywords:** clinical, teaching, students, medical imaging, radiographers

## Abstract

**Introduction:**

Teaching and supervision of medical imaging students are part of the role of many radiographers, yet they are frequently unsupported in the development of their role as a teacher. This study explores radiographers’ experiences and level of confidence in teaching and establishes the areas of support they require to be more effective in their clinical teaching role.

**Methods:**

Sixty radiographers from across Australia completed an anonymous questionnaire, and nine radiographers participated in focus groups. Thematic analysis was conducted on the qualitative data, whilst quantitative data were analysed using one‐way ANOVA and reported as descriptive statistics.

**Results:**

The findings demonstrate that radiographers were mostly confident in the domains of *familiarising students to the practice environment*, *supervising students* and *assisting students to integrate into the practice environment,* but were less confident in *facilitating students’ learning*. Radiographers have identified the teaching skills and attributes they currently possess and the areas in which they need further development.

**Conclusions:**

This study calls for support for radiographers in their teaching role and provides guidance for education providers wanting to design education to support radiographers’ learning needs for teaching.

## Graphical abstract

This article explores radiographers’ experiences and level of confidence in teaching. It establishes the teaching skills and attributes radiographers currently possess and the areas of support they require to be more effective in their clinical teaching role.

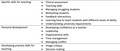

## Introduction

Within clinical settings, effective teaching and supervision are essential for medical imaging (MI) students’ learning. In addition to managing daily workloads, radiographers have an important role as teachers as they guide and support students through authentic learning experiences. Since the early 1990s, inadequate preparation of clinical staff for their teaching role has been highlighted.^1^, ^2^ In addition, confidence in one’s teaching ability is a key driver to engage and contribute to students’ learning experiences.^3^ With little preparation, and limited confidence in a supervisory role, it is understandable that radiographers may be reluctant to take on students, yet supervision is a crucial part of a student’s learning experience.

Currently, little is known about the skills and resources radiographers need to be effective teachers, nor their level of confidence to teach. This study explores radiographers’ experiences of supervision and teaching and identifies areas of low confidence which can be targeted for support to develop capacity in their clinical teaching role.

Teaching and supervising students in clinical settings can be both rewarding and challenging. In a seminal study conducted by Spencer,^4^ challenges of clinical teaching in medicine were identified as time pressures, competing demands, its frequent opportunistic nature (making planning more difficult), increasing numbers of students, fewer patients (due to shorter hospital stays), under‐resourcing, the clinical environment not being ‘teaching friendly’, and poor recognition and rewards for teachers. In the radiation sciences, managing the unpredictability of the clinical environment also creates a challenge for clinical supervisors.^2^ Nonetheless, due to the clinical placement experience being a crucial part of students’ learning experience, it is important that supervisors are supported to take on students.

Despite these challenges, many clinicians do take on the supervision of students and find the experience rewarding. When learning partnerships were formed between radiographers and students, radiographers were motivated to teach by refreshing their knowledge, learning new knowledge and learning from students, enabling them to build their confidence.^3^ When mentors from various health disciplines were surveyed to establish confidence levels in mentoring based on self‐efficacy beliefs, the need for greater confidence in areas such as *assisting students to apply research to practice* and *identifying challenges to the student's learning* was ascertained.^5^ A study of clinical supervisors across health disciplines recommended establishing ‘specific learning needs of particular professional groups and practice settings’ (p. 39),^6^ highlighting the need to ensure learning needs and areas for development are contextualised to both the discipline and context. This study addresses this gap, by exploring radiographers’ experiences and level of confidence of supervision and teaching and the skills and attributes they possess, and those they would like to further develop.

### Conceptual framework

To identify the levels of confidence in teaching and determine the areas in which radiographers need learning support, this study draws on Bandura’s^7^ notion of self‐efficacy, that is, the belief that one’s actions will produce a certain outcome. People with high self‐efficacy are more likely to engage and persist in challenging situations.^8^ For these reasons, understanding radiographers’ levels of confidence in teaching will provide insights into areas to target for professional development in order to increase their confidence and self‐efficacy to foster greater experiences of successful teaching.^9^

## Methods

### Study design and participants

Following ethics approval from Deakin University, chief radiographers at 46 Australian regional and metropolitan clinical sites hosting MI students were contacted and asked to distribute details of the study to their MI staff. Nineteen chief radiographers agreed, and 60 radiographers completed the anonymous questionnaire. Job‐related information was collected and is shown in Table 1. Nine radiographers participated in two focus groups (1 : *n* = 4; 2 : *n* = 5). Participation in all aspects of the study was voluntary.

**Table 1:** Job‐related information for questionnaire participants.

Job‐related information	n (%)
Number of years of practice	
0–5 years	14 (23)
5–10 years	10 (17)
10–20 years	14 (23)
20–30 years	11 (18)
>30 years	11 (18)
Location	
Based at a rural facility	27 (45)
Based at a metropolitan site	33 (55)

NB, Numbers in parenthesis are percentages.

### Questionnaire

Following permission from the primary author, Heale et al.’s^5^ ‘preparation and support for clinical mentors’ questionnaire was adapted for this study. The questionnaire contained 27 items covering four domains (*introducing and familiarising students within the practice environment; supervision; facilitating students’ learning,* and *assisting students to integrate into the practice environment*) that explored radiographers’ level of confidence in relation to various aspects of teaching and supervision on a five‐point Likert scale (1 = Not confident, 2 = Pretty confident, 3 = Usually confident, 4 = Always confident, 5 = Not Applicable). Free‐text fields were included for each domain, and three open‐ended questions asked radiographers to reflect on their current attributes, suggest areas for further development and identify challenges they experienced with teaching and supervision.

### Focus groups

Two semi‐structured focus group interviews were conducted at different locations in Victoria, Australia. Interview questions were designed to explore themes identified in the questionnaire in more detail. Focus groups were audio‐recorded, transcribed verbatim and provided to participants to review and note any errors of fact. Transcripts were de‐identified and pseudonyms used for quotes to protect the anonymity of participants.

### Data analysis

An inductive thematic analysis of qualitative questionnaire and focus group data was conducted in QSR NVivo Pro 11™, guided by Braun and Clarke.^10^ The researchers independently read the transcripts and created coding schemes. These were then discussed collaboratively, refined and final themes agreed upon.

Quantitative Likert scale data were analysed using Microsoft Excel™ and IMB SPSS Statistics for Windows, Version 25 and is are presented as descriptive statistics in frequency tables. Categories of ‘not confident at all’ and ‘pretty confident’ were collapsed into an umbrella category of ‘not confident’, and ‘usually confident’ and ‘mostly confident’ were collapsed into ‘confident’ for ease of discussion within the text.

## Results

A one‐way ANOVA showed no difference in mean confidence ratings across the number of years of practice *F*(4, 55) = 1.057, *P* = 0.386; or by location *F*(1, 58) = 0.006, *P* = 0.941; therefore, results for all participants were analysed as one group.

Table 2 illustrates the combined responses of radiographers across all domains. For Domain 1, results show that most radiographers were confident in *introducing and familiarising students with the practice environment*. Confidence in interpreting protocols, policies and procedures showed the most variability; nonetheless, overall radiographers were generally confident in doing this.

**Table 2:** Radiographers’ level of confidence for teaching and supervision.

How confident are you with:	Not confident at all	Pretty confident	Usually confident	Always confident	NA
Domain 1: Radiographers’ confidence in introducing and familiarising students with the practice environment.					
Familiarising students with the physical environment?	0	1 (2)	9(15)	50 (83)	0
Familiarising students with the practice routine?	0	2 (3)	10 (17)	48 (80)	0
Introducing and interpreting current protocols, policies and procedures to the students?	0	2 (3)	22 (37)	36 (60)	0
Providing an accurate perspective on ‘the way things are done’?	0	3 (5)	15 (25)	42 (70)	0
Providing an accurate perspective on the philosophy of the environment?	0	3 (5)	19 (32)	37 (62)	1 (2)
Average Domain 1	0	4%	25%	71%	0.4%
Domain 2: Radiographers’ confidence in supervision					
Demonstrating current knowledge of clinical practice in medical imaging?	0	3 (5)	19 (32)	38 (63)	0
Demonstrating the ability to organise and prioritise clinical responsibilities?	0	2 (3)	16 (27)	42 (70)	0
Promoting a positive one‐to‐one relationship with students?	2 (3)	1 (2)	22 (37)	35 (58)	0
Understanding the expectations of the educational programme and practice environment?	3 (5)	17 (28)	17 (28)	23 (38)	0
Communicating your expectations clearly to students?	1 (2)	5 (8)	19 (32)	35 (58)	0
Average Domain 2	2%	9%	31%	57%	0%
Domain 3: Radiographers’ confidence in facilitating students’ learning					
Identifying learning needs with students?	4 (7)	8 (13)	32 (53)	16 (27)	0
Providing on‐going, constructive feedback regarding progress?	2 (3)	7 (12)	25 (42)	26 (43)	0
Applying teaching techniques/strategies to facilitate students’ learning?	3 (5)	10 (17)	22 (37)	25 (42)	0
Critiquing images with students?	1 (2)	6 (10)	16 (27)	36 (60)	1(2)
Assisting students to develop their ability to make decisions?*	3 (5)	6 (10)	27 (46)	23 (39)	0
Teaching and role‐modelling professional skills, for example communication?	2 (3)	2 (3)	15 (25)	41 (68)	0
helping students to find suitable learning opportunities?*	3 (5)	5 (8)	23 (39)	27 (46)	1(2)
Stimulating students to apply research to clinical learning situations?*	10 (17)	16 (27)	19 (32)	11 (19)	3(5)
Assisting students to identify strategies for growth and change?	6 (10)	10 (17)	26 (43)	16 (27)	2(3)
Providing opportunities for students to discuss aspects of clinical practice*	1 (2)	9 (15)	24 (41)	25 (42)	0
Assessing the students based on objective standards?	0	11 (18)	21 (35)	26 (43)	2(3)
Average Domain 3	5%	14%	38%	41%	1%
Domain 4: Radiographers’ confidence in assisting students to integrate into the practice environment					
Facilitating students’ collaboration with other members of the healthcare team	1 (2)	6 (10)	22 (37)	30 (50)	1 (2)
Responding to students concerns?	1 (2)	4 (7)	15 (25)	39 (65)	1 (2)
Identifying challenges that are an impediment to the students’ learning?	4 (7)	5 (8)	30 (50)	20 (33)	1 (2)
Resolving challenges that are an impediment to students’ learning?	4 (7)	9 (15)	28 (47)	18 (30)	1 (2)
Consulting appropriate resource persons for assistance when challenges arise?	3 (5)	7 (12)	12 (20)	37 (62)	1 (2)
Resolving conflict situations with students?	4 (7)	5 (8)	27 (45)	15 (25)	9 (15)
Average Domain 4	5%	10%	37%	44%	4%

NB, Numbers in parenthesis are percentages**;** *n = 59 due to missing data.

Regarding Domain 2, whilst most radiographers were generally confident in aspects of *supervision*, many (33%) were less confident in understanding the expectations of the educational programme and practice environment (Table 2).

For Domain 3, *facilitating students’ learning* (Table 2), radiographers reported mixed levels of confidence. Specifically, they were least confident in stimulating students to apply research to clinical learning situations (44%), identifying strategies for students to grow and change (27%) and applying teaching techniques/strategies to facilitate student learning (22%). They were most confident in teaching and role‐modelling professional skills (93%) followed by critiquing images (87%).

Radiographers were generally confident in *assisting students to integrate into the practice environment* (Domain 4, Table 2), in particular responding to students’ concerns (90%); however, they were least confident in resolving challenges that impeded student learning (22%) and consulting appropriate resource persons when challenges arose (17%). Fifteen per cent (15%) of radiographers responded as ‘NA’ to the item of resolving conflict situations with students.

### Qualitative data related to the lived experience of teaching

Two overarching themes of ‘Dimensions of Teaching Practice’ and ‘Teaching Skills and Attributes’ were identified from the focus group and qualitative questionnaire data, which comprised several subthemes. Participants’ names have been replaced by pseudonyms.

#### Theme 1: Dimensions of teaching practice

This theme included subthemes of rewards of teaching, teaching strategies, ways radiographers learned to teach and challenges to teaching.

##### Rewards of teaching

Radiographers highlighted the satisfaction and benefits they experienced from their teaching/supervisory role. They reported learning from the students who contributed to keeping their practice current.

There are so many different techniques, things and exposures that you can find out from a student [from their previous learning experiences] and you can learn a new way to do things, how they do it at this university or this hospital or private clinics (Viv)

….it makes you keep current because you need to make sure you’re teaching the correct things (Lea)

##### Teaching strategies

In their teaching role, radiographers employed various strategies to progress students’ learning. They recognised that different approaches were beneficial to accommodate learning style preferences and radiographers tailored their teaching accordingly.

…. some students learn through being more hands‐on – some students will learn through verbal communication …. so, trying to use a variety of those teaching techniques with each student to figure out which ones they are going to get the most out of (Chris)

A scaffolding approach was employed by radiographers. They were careful not to overwhelm the students, rather they encouraged them to focus on parts of an examination as they gradually worked towards completing an entire examination unassisted.

…not giving them too much at once, … [for]a couple of patients, we’ll just focus on reading their request and understanding how to read a request (Chris)

In their teaching role, radiographers gauged the amount of supervision students needed and emphasised the importance of spending time with students and assessing the patient to get a sense of the level of supervision they required.

I guess it takes time to work with each student to decide, that is, do I need to be with them every step of the way? (Chris)

And then you sort of have to judge, not just the individual student but the patient as well – whether they are a suitable patient (Lea)

##### Learning to teach

A limited number of radiographers reported experiencing formal support for teaching, such as attending workshops related to supervision and teaching. In the absence of such support, radiographers explained that they have learnt to teach by using their experience of being a learner, trial and error and observing the teaching practice of others.

Going back to remembering when I was a student and what sort of methods worked and trying to do the ones that did work (Lilly)

….just observing others…. …when I was doing my placements, I felt like… I’m not going to do that when… or I’ll remember that when I’m qualified (Jess)

##### Challenges to teaching

The availability of time to teach was a significant issue for radiographers. The need to balance competing demands in a busy context, such as the workload and other responsibilities, meant that teaching was constrained by time.

If it’s fairly busy in a certain work area, for example, our CT [computed tomography] department, sometimes you get so busy that you don’t even have time to teach you have to keep the workflow going and …the student just has to stand back and watch everything and kind of learn from a watching experience (Viv)

Radiographers were frequently challenged by the presence of students on short placements and therefore experienced feelings of ‘not knowing’ the students. When students arrived at a new placement, having limited or no knowledge about a student meant it was difficult to ascertain their capability.

Being in a bigger hospital, having students for a short amount of time, or being rostered around, … you might just see them for one day …you don’t have that continuity as far as knowing what they know or where they are at (Lilly)

Sometimes you don’t know what their experience is before, like in a way you want them to have a fresh start at every clinical placement, still sometimes I think if you just had a little bit of background… (Chris)

Developing students’ autonomy was important to radiographers; however, at times they were tested by balancing fostering autonomy and patient care. Although they indicated they would prefer not to ‘take over’, sometimes they intervened to expedite the examination and to ensure patient safety.

You don’t want them to do the wrong thing with the patient, but then also not being right over their shoulder the whole time. I find that hard, I feel like, I’m trying to watch what they’re doing and then sometimes, I want to take over and just quickly do it, …. I need to try and step back and give them time to think and then give them enough space without being intimidating and …. breathing down their neck. So, I think that’s something I find hard (Pene)

Radiographers realised the importance and value of constructive feedback and the need to be direct, but they felt challenged by giving feedback:

If you don’t give that constructive feedback in a way that they [students] understand, they are never going to improve. And I think that’s the thing – you want them to improve – you want them to get better. You’ve got to be nice about it but you can’t butter it up all the time. Like sometimes it needs to be told, particularly when it is around patient safety (Aly)

Like knowing how to phrase things. I do worry what I say to students, …. I usually pull them aside at the end of their placement and just say, what they have done really well or what they maybe need to improve on or work on. (Lilly)

#### Theme 2: Teaching skills and attributes

This theme identifies radiographers’ attributes and skills for teaching and the areas they would like to further develop.

##### Attributes and skills

Radiographers clearly articulated the skills and attributes they currently have for teaching and supervising students as practice experience, strong communication skills (explaining, listening), excellent interpersonal skills (caring, approachable, understanding, empathetic, friendly) and sound knowledge and technical ability.

##### Personal skill development

Specific skills for teaching, supervision and personal development identified by radiographers to support and improve their role as a teacher are outlined in Table 3.

**Table 3:** Areas for further development of radiographers as teachers.

Specific skills for teaching	Clinical assessment Teaching skills Managing struggling students Motivating students Feedback interactions Learning how to teach students with different levels of ability Understanding university requirements
Personal development	Developing confidence as a teacher Leadership Organisational skills Time management Managing conflict
Developing practice skills for teaching	Image critique Decision making

## Discussion

This study explored radiographers’ day‐to‐day practice experiences and levels of confidence in their role as teacher/supervisor of medical imaging students in order to identify areas of support required to build self‐efficacy and become more effective clinical teachers. Effective learning is cultivated when supervisors are provided with the appropriate education rather than relying on teaching students based on their own learning experiences – which may be at odds with contemporary educational practice (e.g. fostering reflection to encourage deeper understanding).^2^, ^11^, ^12^

Contemporary views of higher education focus on developing students to be pro‐active, self‐regulated learners,^13^, ^14^ and radiographers may struggle with supporting this if it contrasts with their own learning experiences which may have been more teacher‐directed. This is also supported by the focus group data, where radiographers described that in the absence of formal teacher training, they tended to teach in the manner in which they learned. In addition to enrolling in online courses or workshops for professional development for teaching support for radiographers, other options may be peer mentoring and developing communities of practice for practitioners involved in teaching.^15^, ^16^ Endorsement for continuing professional development (CPD) could be considered for engagement in such activities.

Consistent with Heale et al.^5^, radiographers also reported not being confident in understanding the expectations of the educational programme and practice environment. Despite universities typically offering advice to clinical staff about expectations of learning for medical imaging students, future research could seek to identify what specific information and guidance radiographers need to increase their understanding of the expectations of the educational programme.

Responses regarding radiographers’ lived experience in both the survey and focus groups centred on the umbrella theme*, dimensions of teaching practice.* This theme included identification of the rewarding aspects of teaching. They learnt from the students who kept them up to date by showing them techniques, skills and knowledge that the students had learned at the university or from other radiographers.

Learning for radiographers also materialised during the act of teaching. For example, radiographers explained that they needed to ensure they maintained their currency in skills and knowledge so that they taught students the correct things. As it is unlikely that radiographers would receive monetary or other extrinsic rewards for the teaching component of their role, intrinsic factors such as interest and enjoyment that are likely to influence engagement in teaching are worthy of further exploration.^17^

Radiographers reported they used various strategies to teach. They emphasised the importance of tailoring or personalising teaching for individuals and recognised the need to understand students’ learning preferences. Scaffolding of learning was fostered by radiographers as students mastered radiographic examinations. Within a cognitive apprenticeship framework, it is during the scaffolding stage that necessary support is provided in the form of suggestions, feedback and reminders. Once the skill has been mastered by the learner, the teacher steps back (fading) and only makes suggestions for the refinement of the skill.^18^ To tailor and introduce a scaffolding approach, radiographers need to know a student’s capabilities. Therefore, adequate time spent with students is needed and maintaining a relationship over time would enable this to happen,^19^ yet time and not knowing a student’s capabilities were identified as challenges by the radiographers in this study.

Radiographers acknowledged that feedback interactions are an essential part of their teacher role, yet this was also seen as a challenge to teaching. They identified guidance in feedback interactions as an area for professional development. Feedback is central to learning, and there is robust evidence in the literature that feedback can have a powerful influence on learning, but its impact is dependent on the type of feedback given and the way it is delivered.^20^, ^21^ The feedback process continues to challenge health professionals. This may be partly due to a traditional unilateral approach to feedback in which a supervisor informs the learner about their performance.^22^ These authors suggest the formation of an ‘educational alliance’ which emphasises the negotiation of goals and dialogue about how to achieve those goals in a committed supervisor–learner relationship. Radiographers are in a prime position to engage in an effective feedback process which Voyer et al.^23^ suggest is supported by three pillars: observation, credibility and the formation of relationships. However, they need support to develop skills and self‐efficacy in this area, as reported above. Radiographers conveyed a lack of confidence in assisting students to identify strategies for growth and change which would be essential parts of a goal‐oriented feedback dialogue.

The findings of this study suggest that *assisting students to integrate into the practice environment* (Domain 4) seems to be a marked challenge for radiographers with a further tension related to knowing how to assist to address challenges, including conflict situations related to students’ learning. It would be interesting to explore why fifteen per cent (15%) of radiographer participants responded to the item of ‘resolving conflict situations with students’ with ‘not applicable’ Does a ‘not applicable’ response suggest that these radiographers have not experienced conflict situations with students? Or do they see that it is not their role to resolve conflict? It does, however, seem pertinent that radiographers are supported to learn how to manage and resolve conflict with students and address challenges related to students’ learning. Other challenges and frustrations radiographers faced in their ‘teacher’ role were inadequate time to teach, including competing demands and other responsibilities. The constraint of time for teaching in clinical settings has been well documented in the literature.^3^, ^4^, ^5^, ^24^ To enable radiographers to jointly teach and meet service requirements, it may be valuable to equip them with efficient teaching frameworks to plan a teaching event, such as the set–dialogue–closure framework described by Peyton.^25^

Radiographers were challenged by insufficient knowledge about a student’s experience and capability when they arrive at a placement. Hence, it is important for students and facilitators to get to know one another, a process described by Paton as ‘artfully connecting’^26^ (p. 145), which involves the supervisor asking the student about relevant personal information to assist in developing rapport, their previous clinical experience and expectations whilst on placement. Further, radiographers focussed on enabling the development of student autonomy, yet they were challenged by attempting to balance the development of student autonomy with patient care and sometimes intervened primarily to ensure patient safety.

The characteristics and attributes that radiographers currently possess for their teaching role were clearly identified by MI participants. Nonetheless, they highlighted the need for additional developmental support and were explicit about their requirements.

### Limitations of the study

It was difficult to establish a response rate for the survey. Chief radiographers agreed to send an invitation to potential participants. However, it was difficult to ascertain the total number of radiographers it was sent to. We do, however, feel confident that we have captured the views and thoughts of a group of radiographers with a wide range of years of experience and a good mix of participants based at both metropolitan and rural facilities.

The findings of this study relied on self‐reported data from participants drawn from clinical sites associated with a regional Australian university and therefore may not be generalisable to other national or international contexts or represent the experiences of those radiographers who chose not to participate. The data have been collected from radiographers within the state of Victoria, Australia. Radiographers may report different experiences of supervision in other Australian states due to different supervision requirements. However, the qualitative data revealed trends in experiences, and consistent themes were identified across the questionnaire and focus group comments providing rigour to the authors’ findings and recommendations.

### Recommendations

Education providers could consider a variety of initiatives to support radiographers’ professional development as teachers (Table 4).

**Table 4:** Recommendations to support professional development for radiographers’ teaching role.

A combination of approaches (with potential consideration of endorsement for continuing professional development)	Workshops Online initiatives (e.g. an engaging online resource offering support and guidance to radiographers in their teaching/supervisory role)
A peer‐mentoring teach‐the teacher scheme	Mentoring relationships formed between experienced and less experienced radiographers who teach students
Develop ‘communities of practice’	To enable radiographers to share experiences of supervision and teaching

## Conclusion

Radiographers have articulated varied experiences of supervising and teaching students. They have highlighted the rewards of teaching and identified challenges they face in their teaching/supervisory role. Radiographers have openly expressed both the skills and attributes they currently possess for teaching and their needs for further development. The findings of this study challenge education providers to offer one or more initiatives (as recommended above) to support a radiographer's teaching role. Guidance for teaching may foster radiographers’ self‐efficacy beliefs resulting in greater confidence and success in their teaching. Initiatives to support the development of radiographers as teachers ought to be considered for approval for continuing professional development requirements.
